# Characteristics of the stressors associated with suicidal behavior in adolescence

**DOI:** 10.1192/j.eurpsy.2024.966

**Published:** 2024-08-27

**Authors:** S. Susana Perez, I. Martin

**Affiliations:** ^1^Psychiatry, Los Arcos del Mar Menor Hospital; ^2^Psychiatry, Infante-Murcia Mental Health Center, Murcia, Spain

## Abstract

**Introduction:**

In the assessment of suicidal behavior, recent studies describe the great influence of an environmental component with adverse life events and stressors that can influence ideation and self-harm.

**Objectives:**

-1. We propose to analyze the reasons for consultation of adolescents between 12 and 16 years old who consult for suicidal ideation/behavior. 2. Estimate the frequency of different socio-family life events.

**Methods:**

-A retrospective review of emergency consultations in the last 4 months is performed. Sociodemographic data, vital events, reason for consultation and evolution in the following 40 days after the first consultation are collected.

**Results:**

-Data are collected from 16 adolescents who consult due to suicidal ideation/gesture in a period of 4 months, of which 42% (7) are women and 57% (9) are men. The reasons recorded as stressful life events were: 32% unstructured family environment, 13% death of a close relative, 37% poor parental supervision, 26% end of a romantic relationship, 15% legal problems, 2% sexual or physical abuse, 68 % academic problems, 13% bullying. It was observed that in 63% of the cases they had more than one adverse experience.

**Conclusions:**

-Different adverse life events frequently precede suicidal ideation and behavior that can be minimized or go unnoticed and undervalued. A meticulous clinical history can clarify some of the reasons that influence the hopelessness and clinical anguish that suicidal patients present. Its early detection provides the opportunity for an early and specialized approach

**Disclosure of Interest:**

None Declared

